# Reliability of Handgrip Strength Test in Basketball Players

**DOI:** 10.2478/v10078-012-0003-y

**Published:** 2012-04-03

**Authors:** Vassilis Gerodimos

**Affiliations:** 1 Department of Physical Education and Sport Sciences, University of Thessaly, Trikala, Greece

**Keywords:** test-retest, isometric strength, hand preference, age, children

## Abstract

Handgrip strength is important in basketball as various movements rely on the continuous use of wrist and digits flexor muscles when catching, holding, shooting and throwing the ball. Therefore, the assessment of handgrip strength is used in prepubertal, adolescent and adult basketball players. The reliability of handgrip strength may be influenced by several factors including age. The purpose of this study was to examine the reliability of handgrip strength in basketball players from childhood to adulthood. Male basketball players (n = 90) were assigned into three groups: prepubertal (9.8 ± 0.7yrs), adolescents (14.4 ± 0.6yrs), and adults (26.1 ± 5.6yrs). Each participant performed three maximal isometric contractions on each hand in two occasions, one day apart. Intraclass correlation coefficient (ICC), standard error of measurement (SEM) and 95% limits of agreement (LOA) were calculated. The test-retest reliability was high for both preferred (ICC = 0.94 – 0.98) and non-preferred (ICC = 0.96 – 0.98) hands, without differences in reliability among age-groups. The results showed a significant age-related increase (p < 0.05) in absolute and relative handgrip strength irrespective of hand preference. The present results indicate that maximum handgrip strength can be measured reliably, using the Jamar hand dynamometer, in basketball players from childhood to adulthood.

## Introduction

Handgrip strength is important in basketball as various movements rely on the continuous use of wrist and digits flexors in catching, holding, shooting and throwing the ball (Cortis et al., 2011; Visnapuu et al., 2007). The evaluation of handgrip strength is often used in basketball, since hand dynamometry is simple, not expensive, and a well-established method for assessing the strength of wrist and digits flexor muscles.

Reliability is an important aspect of strength testing protocols. The reliability of measurement is influenced by several factors such as the type of test, training status, gender, duration of test (Hopkins et al., 2001). In basketball, although several studies examined handgrip strength in young and adult players (Angyan et al., 2003; Cortis et al., 2011; Coelho e Silva et al., 2008; 2010; Visnapuu et al., 2007), only two studies examined the reliability of the handgrip strength test. Coelho e Silva et al. (2008; 2010) reported high reliability of the handgrip strength test in young adolescent basketball players (14–15.9 years-old and 12–13.9 years-old, respectively). There is evidence that the reliability of strength measurements may be influenced by age. To the best of our knowledge, no previous study has examined the reliability of the handgrip strength test in prepubertal basketball players. The reliable evaluation of handgrip strength in pubertal basketball players is an essential component in strength monitoring, in planning of strength training programs, as well as in injury prevention and recovery.

There are several studies that have examined the reliability of the handgrip strength test in untrained children (Espana-Romero et al., 2008), adolescents (Clerke et al., 2005; Espana-Romero et al., 2010b; Ortega et al., 2008; Ruiz et al., 2006) and adults (Lagerstrom et al., 1998; Peolsson et al., 2001; Ruiz-Ruiz et al., 2002; Shechtman et al., 2005). Espana-Romero et al. (2008) and Clerke et al. (2005) reported high test-retest reliability of handgrip strength in children and adolescent males, respectively. In addition, Peolsson et al. (2001) and Ruiz-Ruiz et al. (2002) found high reliability of the handgrip strength test in healthy adults using the Jamar and Takey dynamometers, respectively.

The reliability of handgrip strength could be influenced by age. Differences in mood, motivation, learning effect, the ability to focus on the task, as well as biomechanical factors such as hand size may account for these age-related differences in reliability (Molenaar et al., 2008; Svensson et al., 2008). The few studies that examined the reliability of the handgrip strength test, in untrained participants, at different age-groups demonstrated equivocal findings. Espana-Romero et al. (2010a) found high reliability of handgrip strength in both children and adolescents, using the Takey hand dynamometer. In addition, Molenaar et al. (2008) examined the reliability of handgrip strength among three age groups of children (4–6, 7–9, and 10–12 years-old), and found no clear age-effect on reliability for both dynamometers (Lode dynamometer and Martin vigorimeter) that have been used in the study. Contrary, Svensson et al. (2008) compared the reliability of handgrip strength test among 6, 10 and 14 years-old children using the Grippit dynamometer, and found greater reliability in 6 and 14 years-old compared to the 10 year-old children. It should be pointed out that the aforementioned studies have been performed in untrained participants. Nevertheless, the reliability of the test may be influenced by training status (Hopkins et al., 2001).

To the best of our knowledge no study has examined the reliability of the handgrip strength test in basketball players throughout the developmental ages. Therefore, the aim of this study was to examine the test-retest reliability of the handgrip strength test in prepubertal, adolescent and adult male basketball players.

## Methods

### Participants

Ninety male basketball players volunteered to participate in the present study. Following a completion of a medical history form, the participants were divided according to their chronological age into three groups: prepubertal (9.8 ± 0.7 yrs), adolescents (14.4 ± 0.6 yrs), and adults (26.1 ± 5.6 yrs). All participants were healthy and had no previous injury of upper limbs. Before the start of the study, the institutional Ethics Committee approved the experimental protocol. Next, the adult participants and the parents of prepubertal and adolescents signed a written informed consent form. The anthropometrical and training characteristics of the subjects are presented in Table 1.

### Testing Procedures

Each participant reported to the exercise laboratory in the morning of the testing. Following orientation, anthropometrical characteristics (body height and body mass), pubertal stage and hand preference were determined before the testing session.

The pubertal stage was determined according to pubic hair development (Tanner et al., 1976). Hand preference was determined by asking the participant of which hand is used to hold a pencil.

Next, the participant performed a standardized warm-up that included two to three preliminary trials for familiarization with the recording procedure and instrumentation. A portable digital hand dynamometer (Jamar, EN-120604), was used for handgrip strength measurement. The test was performed in the sitting position with the shoulder of tested arm adducted, the elbow flexed at 90°, whereas the forearm and wrist were set in neutral position (De Smet et al., 2001; Holm et al., 2008). The testing protocol consisted of three maximal isometric contractions for 5 s, on both hands, with a rest period of at least 60 s and the highest value was used for determination of maximal grip strength. The subjects were instructed to squeeze the dynamometer as hard as possible. Visual feedback of the recorded strength was provided. The parameters used for analysis were: peak absolute strength (kg) and relative handgrip strength (kg/kg of body mass). The following day the procedure was repeated at the same time of the day, location, and instructions. The order of testing the “preferred” and the “not preferred” hands on days 1 and 2 was randomized to avoid cross-over effects.

#### Data Analysis

Test-retest data was analysed using the Intraclass Correlation Coefficient (ICC). The ICC value varies between 0 and 1, where an ICC of 0 indicates no reliability, while an ICC value equal to 1 indicates perfect reliability. An ICC value equal or greater than 0.80 is considered high. We calculated ICC for single measures using a twoway random effect model of absolute agreement for the computation of ICC. Although the ICC is a well-accepted measure of relative reliability, it is difficult to interpret ICC values since they are highly depended on the variability of the group being assessed. In order to assess the absolute reliability, the standard error of measurement (SEM) and the 95% limits of agreement (LOA) were calculated by means of the following equations: SEM = SD x (1 - ICC)^0,5^ and LOA = inter-trials mean difference ± 1.96 SD of the inter-trials difference (Atkinson et al., 1998). The SEM expresses measurement error in the same units as the original measurement, and it is not influenced by variability among patients. The SEM was divided by the mean of the two measurements and multiplied by 100 to give a percentage value (SEM %; Svensson et al., 2008). The inter-trials agreement was also examined graphically by plotting the difference between test and retest against their mean, according to the Bland and Altman approach (Bland and Altman, 1986). The presence of heteroscedasticity was tested using the Pearson correlation test, in order to examine whether the absolute inter-trial difference associated with the magnitude of the measurement. A two-way analysis of variance (ANOVA; age × time) with repeated measures on the “time” factor was used to determine possible differences in handgrip strength between test and retest.

A one-way ANOVA for independent groups was used to examine the effect of age on anthropometrical and training characteristics. A two-way analysis of variance (ANOVA; age × hand) with repeated measures on the “hand” factor was used to analyze the effects of age and hand preference on grip strength. Tukey’s post-hoc tests were used to locate the significantly different means. The level of significance was *p* < 0.05. All data are presented as Means ± SD and were analyzed using SPSS 13.0 (Illinois, USA).

## Results

### Reliability

**Figure 1:**
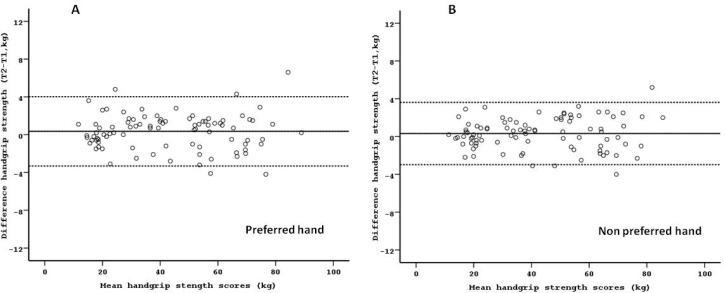
Bland-Altman plots of the handgrip strength test for preferred and non-preferred hand in the whole group. The central line characterizes the mean difference between test and retest values; the upper and lower lines characterize the upper and lower 95 % limits of agreement (LOA = inter-trials mean difference ± 1.96 SD of the inter-trials difference), respectively

Initially, the data was analyzed independent of age (whole sample). The ANOVA results indicated non-significant differences between test and retest handgrip strength values. The relative reliability between test and retest was very high. The ICC ranged from 0.994 to 0.997 for the preferred hand and from 0.995 to 0.998 for the non-preferred hand. The absolute reliability (SEM and LOA) was good. The mean absolute difference between test and retest was 0.35 kg and 0.32 kg for the preferred and non-preferred hands, respectively. Whereas, the 95 % limits of agreement ranged from −3.32 kg to 4.02 kg for the preferred hand and from −2.97 kg to 3.61 kg for the non-preferred hand (Figure 1). No presence of heteroscedasticity was observed. Test and retest data, as well as relative and absolute reliability measures are presented in Table 2.

**Figure 2:**
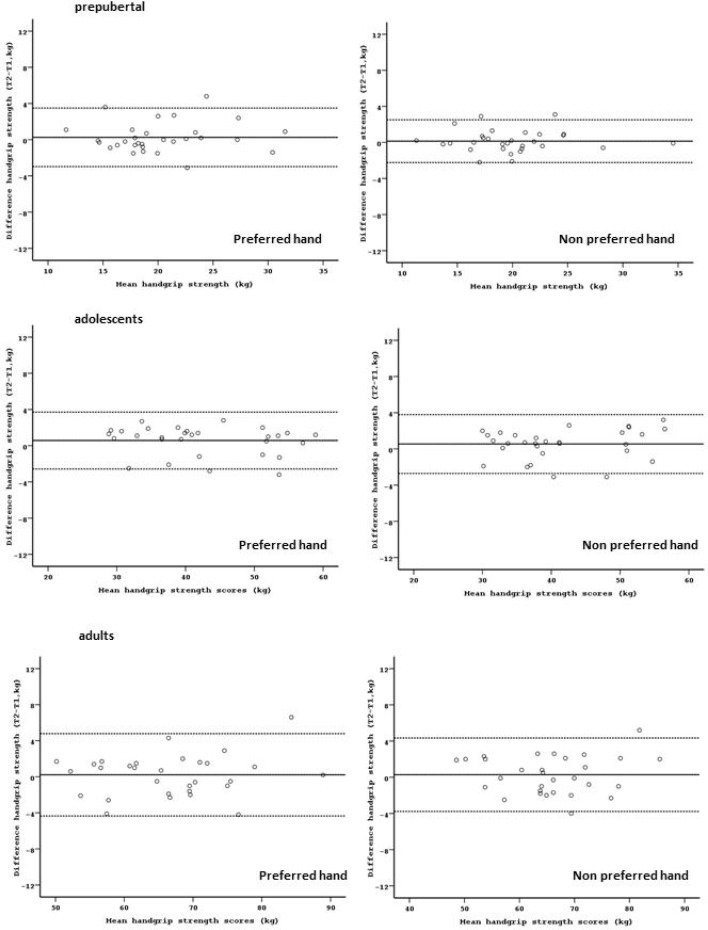
Bland-Altman plots of the handgrip strength test for preferred and non-preferred hand in each age group. The central line characterizes the mean difference between test and retest values; the upper and lower lines characterize the upper and lower 95 % limits of agreement (LOA = inter-trials mean difference ± 1.96SD of the inter-trials difference), respectively

For the non-preferred hand the ICC was 0.966, 0.98, and 0.975 in prepubertal, adolescents and adults, respectively. The absolute reliability (SEM and LOA) was good in the three age groups. No presence of heteroscedasticity was observed (Figure 2). The test and retest data as well as ICC, SEM and LOA in each group are presented in Table 2.

### Grip strength: effect of age and hand

**Figure 3:**
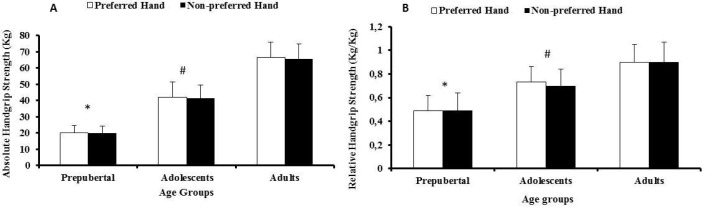
Absolute (A) and relative (B) handgrip strength values (kg; kg / kg BM, respectively) in children, adolescents, and adults basketball players. Values are means ± SD in each hand per age group; *p < 0.05 vs. all other groups; #p < 0.05 vs. adults.

The values obtained from the first day were used to examine the effect of “age” and “hand” on grip strength. The peak absolute and relative handgrip strength values across the age groups in each hand are presented in Figure 3. Although peak absolute and relative handgrip strength significantly increased across the age-groups (*p* < 0.05), no significant differences between the “preferred” vs. “non-preferred” hand were observed

## Discussion

The results of this study demonstrated that handgrip strength, using the Jamar dynamometer, can be measured reliably in prepubertal, adolescent and adult male basketball players. No significant age differences, in reliability of handgrip strength test were observed for both preferred and non-preferred hands. In addition, our data revealed that both absolute and relative handgrip strength increased during the developmental years in basketball players. However, there were no significant differences, in both absolute and relative handgrip strength between preferred and non-preferred hands.

### Reliability

The findings of the present study are in line with previous studies (Table 3) that reported high reliability of handgrip strength in untrained children (Espana-Romero et al., 2008), adolescents (Espana-Romero et al., 2010b; Ruiz et al., 2006) and adults (Langerstrom et al., 1998; Peolsson et al., 2001; Ruiz-Ruiz et al., 2002; Shechtman et al., 2005) using different types of hand dynamometers. Particularly, Espana-Romero et al. (2008) reported high reliability (ICC = 0.97 – 0.98) of the handgrip strength test in 6–12 year-old children, using the Takey dynamometer. Excellent test-retest reliability (r = 0.96 – 0.98) of handgrip strength have been also showed in untrained adolescents (14–17 years-old; Ruiz et al., 2006). In addition, Langerstrom et al. (1998) and Ruiz-Ruiz et al. (2002) found high reliability (r = 0.91 – 0.97) of the handgrip strength test in healthy adults using the Grippit and Takei dynamometers, respectively. The results of this study are also, in accordance with those by Coelho e Silva et al. (2008; 2010) in young basketball players (14–15.9 years-old and 12–13.9 years-old, respectively) that reported high reliability (r = 0.99) of handgrip strength using the Lafayette hand dynamometer.

Our results support earlier findings that showed non-significant differences in handgrip strength between test and retest values (Espana-Romero et al., 2008; 2010a). In contrast, Clerke et al. (2005) found small but significant differences in handgrip strength between test and retest, in 13 to 17 year-old adolescents. The absence of warm-up or familiarization prior to testing in the above study may account for the differences in handgrip strength between test and retest measurements. Indeed, Svensson et al. (2008), who also found differences in handgrip strength between test and retest suggested that children may learn over the trials a better technique or accomplish to squeeze harder. Therefore, the authors recommended a familiarization session and three maximal trials during the main testing.

### Reliability and age-effect

Only a few studies addressed the issue of age-effect on reliability of handgrip strength in untrained participants (Table 4). The results of our study are in line with those of Espana-Romero et al. (2010a) who examined the reliability of the handgrip strength test in untrained children (6–11 years-old) and adolescents (12–18 years-old) using the Takey dynamometer and found high reliability in both age-groups. Moreover, Molenaar et al. (2008) compared the reliability of handgrip strength among three age-groups of untrained children (4–6, 7–9, and 10–12 years old) using two different dynamometers (Lode dynamometer vs. Martin vigorimeter), and reported no clear age-effect on reliability for both dynamometers.

In contrast, Svensson et al. (2008) compared the reliability of the handgrip strength test among 6, 10 and 14 year old untrained children using the Grippit dynamometer, and showed greater reliability in 6 and 14 year old (ICC = 0.96) compared to 10 year old children (ICC = 0.78). The authors suggested that the age-related differences in reliability may be due to differences in mood, motivation, concentration between test and retest or biomechanical factors such as hand size in relation to handle size.

#### Grip strength: effect of age and hand

Prepubertal and adolescents in this study exhibited relatively similar peak handgrip strength values to those previously reported in their peers of the general population (De Smet et al., 2001; Hager-Ross et al., 2002; Holm et al., 2008). However, adult basketball players in this study demonstrated higher peak hand grip strength values than those previously reported in untrained adult males (Gunther et al., 2008; Massy-Westropp et al., 2004). These differences are explained by the training stimulus exerted in basketball, since grip strength is influenced by physical activity level and training (Gojanovic et al., 2009; Nevill, 2000).

The gradually increasing values in handgrip strength from childhood to adulthood observed in this study, are in accordance with previous findings demonstrating an age-related increase in grip strength in untrained and trained boys during growth and development (De Smet et al., 2001; Hager-Ross et al., 2002; Molenaar et al., 2010; Visnapuu et al., 2007; 2009).

In this study, male basketball players of all age-groups exhibited non-significant differences in grip strength between preferred and non-preferred hand.

Previous studies that examined handgrip strength during the developmental years found either hand-related differences in right-handed children (Hager-Ross et al., 2002) and adolescents (Clerke et al., 2005), or no effect of hand preference in untrained participants (De Smet et al., 2001). In athletes, Gojanovic et al. (2009) in tennis players and Margonato et al. (1994) in fencers, observed significant differences between the dominant and the non-dominant hand. These hand-related differences in athletes may be due to the asymmetrical training of the dominant hand in these sports. Basketball, however, includes the continuous use of both hands in catching, holding, dribbling and passing the ball which explains the lack of differences in handgrip strength between the dominant and the non-dominant hand in our study.

## Conclusions

The reliability of handgrip strength, using the Jamar dynamometer, is high in prepubertal, adolescent and adult male basketball players. There were no significant effects of age and hand on reliability of the handgrip strength test. Maximal absolute and relative handgrip strength gradually increases from childhood to adulthood in basketball players. Finally, no significant differences were observed in handgrip strength, between preferred and non-preferred hands possibly due to the continuous use of both hands in basketball. This study has established a reliable testing protocol for the evaluation of handgrip strength and provides normative data of peak absolute and relative handgrip strength in prepubertal, adolescents and adults basketball players that can be used for strength monitoring and planning of strength training programs. Currently, it is not known whether the specificity of basketball training and/or the different use of wrist and digits flexor muscles in basketball players may affect the generalization of our results to athletes of other sports. There is, however, a general believe that the reliability of strength measurements and/or the normative values vary when examining a population with different characteristics (e.g. children vs. adults, untrained vs. trained). Thus, future studies should establish a reliable handgrip strength test and norms for athletes of other sport disciplines.

## Figures and Tables

**Table 1:** *Anthropometrical* and training characteristics of the participants (Mean ± SD)

	Age groups		
Variables	Prepubertal (n = 30)	Adolescents (n = 30)	Adults (n = 30)
Age (yrs)	9.85 ± 0.70^*^	14.37 ± 0.61^#^	26.06 ± 5.57
Maturity (stage)	1–2	3–4	5
Body height (cm)	145.59 ± 8.33^*^	172.63 ± 9.76^#^	193.23 ± 8.19
Body mass (kg)	42.36 ± 10.12^*^	66.85 ± 13.61^#^	96.60 ± 15.91
Training experience (yrs)	1.99 ± 1.39	3.52 ± 1.67	12.69 ± 6.82^*^

* p<.05 vs. all other groups;

# p<.05 vs. adults

**Table 2:** Test and retest values, and index of relative and absolute reliability of handgrip strength in each group.

Age groups	Test (kg)	Retest (kg)	Bias (kg)	ICC (95% CI)	95% LOA (kg)		SEM (kg)	SEM %
					Lower	Upper		
Whole group (n =90)								
*Preferred hand*	42.88±20.69	43.23±20.69	0.35 ± 1.87	0.996 (0.994–0.997)	−3.32	4.02	1.24	2.88
*Non-preferred hand*	42.24 ±20.32	42.56±20.42	0.32 ± 1.68	0.997 (0.995–0.998)	−2.97	3.61	1.02	2.41

Prepubertal (n = 30)								
*Preferred hand*	20.06 ± 4.67	20.32±4.81	0.26 ± 1.65	0.940 (0.879–0.971)	−2.97	3.49	1.12	5.55
*Non-preferred hand*	19.78 ± 4.59	19.92±4.59	0.14 ± 1.21	0.966 (0.930–0.984)	−2.23	2.51	0.82	4.13

Adolescents (n = 30)								
*Preferred hand*	42.10 ± 9.44	42.67±9.15	0.57 ± 1.60	0.984 (0.965–0.992)	−2.57	3.71	1.20	2.83
*Non-preferred hand*	41.27 ± 8.41	41.81±8.73	0.54 ± 1.66	0.980 (0.957–0.990)	−2.71	3.79	1.19	2.86

Adults (n = 30)								
*Preferred hand*	66.49 ± 9.33	66.71±9.68	0.22 ± 2.33	0.971 (0.940–0.986)	−4.35	4.79	1.60	2.40
*Non-preferred hand*	65.68 ± 9.16	65.95±9.26	0.28 ± 2.07	0.975 (0.949–0.988)	−3.78	4.34	1.46	2.22

Bias: difference between test and retest, ICC: intraclass correlation coefficient, 95 % CI: 95 % confidence interval, 95 % LOA: 95 % limits of agreement, SEM: standard error of measurement, SEM %: standard error of measurement expressed as a percentage value

**Table 3:** Test-retest reliability of maximal handgrip strength in healthy children, adolescents and adults

Author	Participants	Protocol/Instrument	Statistical Methods	Main Outcome
Clerke et al. (2005)	♂=74, ♀=75 (13 – 17yrs)	sitting position, elbow (90°), 1 trial, H: DH & NDH, HD: GripTrack	ICC_3,1_, SEM, ANOVA	test-retest (↑0.62 kg), ICC_3,1_= 0.954–0.973 (♂) & 0.920-0.476 (♀), SEM=1.83–2.75 kg (♂) & 1.63–2.58 kg (♀)
Coelho E Silva et al. (2008)	21♂BP (14 – 15.9yrs)	best of 2 trials, HD: Lafayette	r, σ_e_	r = 0.99, σ_e_ = 0.9 kg
Coelho E Silva et al. (2010)	21♂BP (12 – 13.9yrs)	best of 2 trials, HD: Lafayette	r, σ_e_	r = 0.99, σ_e_ = 0.9 kg
Espana-Romero et al. (2008)	♂=17, ♀=5 (6 – 12yrs)	standing position, elbow extended, optimal grip span, H: RH & LH, HD: Takei	r, ANOVA	r= 0.972 (RH) & 0.985 (LH), test-retest NS
Lagerstrom et al. (1998)	♂=4, ♀=25 (28 – 71yrs; 49 ± 8.9yrs)	sitting position, elbow (75–85°), last 3 trials, H: DH & NDH, HD: Grippit	ICC, C_R_, CV, ANOVA	*test-retest:* test-retest NS, DH<NDH, r=0.91–0.96, CR=47.2–53.2, CV: 5.6–6.9%
Ortega et al. (2008)	69♂ & 54♀ (13.6 ± 0.8 yrs)	optimal grip span, H: RH & LH, HD: Takey	bias, ANOVA, Bland-Altman plots (95% LOA)	test-retest NS, ♂ & ♀ NS bias: 0.3±2.5 kg (♂) & 0.0±1.8 kg (♀)
Peolsson et al. (2001)	8♂ & 24♀ (20–64 yrs; 29 ± 10 yrs)	standing position, elbow (90°), GP:2 (♀) & 3 (♂), H: RH & LH, HD: Jamar	ICC, ANOVA	*Intra-tester reliability* ICC: 0.98 (RH) & 0.94 (LH) *Inter-tester reliability* ICC: 0.98 (RH & LH)
Ruiz et al. (2006)	13♂ & 4♀ (14–17 yrs)	standing position, elbow extended, optimal grip span, H: RH & LH, HD: Takey	r, ANOVA	r=0.98 (RH) & 0.96 (LH), test-retest NS
Ruiz-Ruiz et al. (2002)	5♂ & 5♀ (20–80yrs)	standing position, elbow extended, optimal grip span, H: RH & LH, HD: Takey	r, paired t-test	r=0.96 (LH) & 0.97 (RH), test-retest NS
Shechtman et al. (2005)	50♂ & 50♀ (20–40yrs;23.5±3. 5yrs)	sitting position, elbow (90°), GP: 2, mean of 3 trials, H: RH & LH, HD: DynEx & Jamar	r, CV%	*Study with human subjects* r=0.9864 (DynEx) & r=0.9856 (Jamar) *Study with known weights* r=0.999 (DynEx) & r=0.999 (Jamar) CV: 1.63% (DynEx) & 7.74% (Jamar), DynEx<Jamar

♂:*males,* ♀*:females, H: hand, DH: dominand hand, NDH: non-dominand hand, HD: hand dynamometer, ICC: intraclass correlation coefficient, SEM: standard error of measurement,* ↑*: significant increase, BP: basketball players, r: reliability coefficient, σe: technical error of measurement, RH: right hand, LH: left hand, h: hours, NS: not-significant differences, CR: coefficient of repeatability, CV: coefficient of variation, 95%LOA: 95% limits of agreement, GP: grip position, <: lower measurement error.*

**Table 4:** Test-retest reliability of maximal handgrip strength at different age-group.

Author	Participants	Protocol/Instrument	Main Outcome
Espana-Romero et al. (2010a)	138: ♂ & ♀ Groups: G1(6–11.9yrs) G2(12–18 yrs)	standing position, elbow extended, GP: adjusted, best of 2 trials, H: RH & LH, HD: Takey	test-retest NS in both G1 & G2 G1: SSE= 68.91kg, MSE= 1.28kg, RMSE= 1.13kg, %Error= 2.48, SEE= 1.13kg G2: SSE= 430.40kg, MSE = 5.66kg, RMSE=2.38kg, %Error=3.43, SEE= 2.38kg
Molenaar et al. (2008)	♂=45, ♀=59 Groups: G1 (4–6 yrs) G2 (7–9 yrs) G3 (10–12yrs)	sitting position, elbow (90°), GP: 2 (LD) & medium bulb (MV), mean of 3 trials, H: DH & NDH, HD: LD & MV	LD>MV, test-retest NS, SDD%: ≠G1, G2, G3 *Lode dynamometer* G1: ICC =0.73–0.91 (0.50–0.96), SEM=6.7–7.9N, SDD = 18.4–22N, SDD% = 27.6 – 35.5, G2: ICC = 0.78–0.79 (0.62–0.88), SEM = 10.3–12.2N, SDD=28.4–33.9N, SDD%=25.6–28.5 G3: ICC=0.82–0.92 (0.66–0.98), SEM=11.3–14.6N, SDD=31.2–40.5N, SDD%=16.9–23.2 *Martin vigorimeter* G1: ICC=0.76–0.79 (0.55–0.90), SEM=4.6–4.9kPa, SDD = 12.6–13.5kPa, SDD%=33.6–34.9 G2: ICC = 0.47–0.55 (0.17–0.74), SEM =5.3–5.7kPa, SDD=14.7–15.7kPa, SDD%=26.9–28.7 G3: ICC=0.70 (0.48–0.84), SEM=7.5–7.6kPa, SDD=20.8–21.1kPa, SDD%=30.8–31.1
Svensson et al. (2008)	♂=26, ♀=32 Groups: G1 (6yrs) G2 (10yrs) G3 (14yrs)	sitting position, elbow (90°), GP: adjusted, best of 3 & mean of 3 trials, H: DH & NDH, HD: Grippit	G2<G1 & G3, test-retest NS, best of 3>mean of 3 *Best of three* G1: ICC=0.96 (0.90–0.99), SEM=4.8N, SEM%:6.3, CR:13.3N G2: ICC=0.78 (0.54–0.91), SEM=20.1N, SEM%:12.5, CR:55.7N G3: ICC=0.96 (0.90–0.98), SEM=16.8N, SEM%:5.2, CR:46.7N *Mean of three* G1: ICC=0.96 (0.89–0.98), SEM=4.7N G2: ICC=0.74 (0.46–0.89), SEM=20.9N G3: ICC=0.93 (0.83–0.97), SEM=19.4N

♂: males, ♀: females, G1: group 1, G2: group 2, GP: grip position, H: hand, RH: right hand, LH: left hand, HD: hand dynamometer, NS: not significant, SSE: sum of squared errors, MSE: mean sum of squared errors, RMSE: root mean sum of squared errors, % error: the percentage error, SEE: standard error of estimate, G3: group 3, LD: Lode dynamometer, MV: Martin vigorimeter, DH: dominand hand, NDH: non dominand hand, ICC: intraclass correlation coefficient, 95%CI: 95% confidence interval, SEM: standard error of measurement, SDD: smallest detectable difference, SDD%: smallest detectable difference expressed as a % Maximal voluntary contraction, >: higher reliability, ≠: different, SEM%: standard error of measurement expressed as a percentage value, CR: coefficient of repeatability, <: lower reliability, >: higher reliability.
